# PERSON-FIRST LANGUAGE FOR OLDER ADULTS INCARCERATED IN PRISON: A RAPID EVIDENCE ASSESSMENT

**DOI:** 10.1093/geroni/igad104.3522

**Published:** 2023-12-21

**Authors:** Kristin Merss, Laura Block, Stephen Kwas

**Affiliations:** University of Wisconsin - Madison, School of Nursing, Madison, Wisconsin, United States; University of Wisconsin-Madison, Madison, Wisconsin, United States; Swarthmore College, Madison, Wisconsin, United States

## Abstract

Language can influence a person’s sense of self, relationship with healthcare providers, healthcare utilization, or their own symptoms and behaviors. The person-first language movement began decades ago and has been widely adopted by multiple fields including nursing, medicine, gerontology, and social and health sciences more broadly. For research involving older adults, non-stigmatizing language has become the standard for researchers. Incarceration is widely considered a dehumanizing experience, with recent calls for attention to the language used by researchers when describing their incarcerated participants. Researchers are also identifying that individuals with multiple stigmatizing identities (e.g., incarcerated and disabled, older adult) warrant specific consideration. This study presents a secondary review of terminology from a Rapid Evidence Assessment (REA) investigating what is known about transitions of older adults in the incarceration system. The REA included PubMED, PscyhMED, and CINHAL. Nineteen articles met inclusion criteria (11 qualitative, 11 quantitative, 3 mixed methods). As a sub-analysis, articles were analyzed to consider the language that was used to describe the currently or formerly incarcerated older adults who were the subject of the studies. The majority of articles considered older adults who were no longer incarcerated. While person-first language (i.e., “formerly incarcerated person”, “reintegrating women”, “older adult on parole”) was common, stigmatizing language such as “prisoner”, “offender”, “homicide offender”, and “ex-convict” were used in the majority of the studies included in the review. Appreciation and adoption of person-first, non-stigmatizing language should transcend not only research literature surrounding older adults in the community, but also incarcerated older adults.

